# Motivational Determinants of Exergame Participation for Older People in Assisted Living Facilities: Mixed-Methods Study

**DOI:** 10.2196/jmir.6841

**Published:** 2017-07-06

**Authors:** Wytske Meekes, Emma Kate Stanmore

**Affiliations:** ^1^ Division of Nursing, Midwifery and Social Work and MAHSC (Manchester Academic Health Science Centre) Manchester United Kingdom

**Keywords:** technology, aged, accidental falls, rehabilitation, motivation

## Abstract

**Background:**

Exergames (exercise-based videogames) for delivering strength and balance exercise for older people are growing in popularity with the emergence of new Kinect-based technologies; however, little is known about the factors affecting their uptake and usage by older people.

**Objective:**

The aim of this study was to determine the factors that may influence the motivation of older people to use exergames to improve their physical function and reduce fall risk.

**Methods:**

Mixed methods were employed in which 14 semistructured interviews were conducted with older people (n=12, aged 59-91 years) from 2 assisted living facilities in the North West of the United Kingdom. The older people participated in a 6-week trial of exergames along with one manager and one physiotherapist; 81 h of observation and Technology Acceptance Model questionnaires were conducted.

**Results:**

The findings suggest that the participants were intrinsically motivated to participate in the exergames because of the enjoyment experienced when playing the exergames and perceived improvements in their physical and mental health and social confidence. The social interaction provided in this study was an important extrinsic motivator that increased the intrinsic motivation to adhere to the exergame program.

**Conclusions:**

The findings of this study suggest that exergames may be a promising tool for delivering falls prevention exercises and increasing adherence to exercise in older people. Understanding the motivation of older people to use exergames may assist in the process of implementation.

## Introduction

The proportion of older people in the world population is increasing as never before [1-3]. Older people are at increased risk of falls due to factors such as impaired balance, gait problems, poor muscle strength, visual impairment, psychotropic medications, multiple drug use, impaired cognition, and urinary incontinence [4,5]. Approximately 28-35% of people aged 65 years and older fall each year [6]. The consequences of a fall can vary: from a bruise or sprain to traumatic brain injury, hip fracture, or even death [6,7]. These consequences can lead to decreased health and well-being and increased health care sector costs [6]. One of the best modifiable risk factors to reduce fall risk among older people is physical exercise, in particular, specific strength and balance exercises [8]. However, motivation to engage in physical activity is often low in old age.

A potential method to increase physical activity may be the use of exergames. Exergames are videogames that combine gameplay with physical exercise. This paper describes the use of MIRA-exergames [9,10] that were developed with older people, academics, and 2 falls prevention teams (including physiotherapists, geriatricians, occupational therapists, and rehabilitation nurses) for older people to improve their physical function and reduce fall risk. The MIRA system [9] uses Microsoft Kinect, an off-the-shelf 3D camera motion tracking device that can track the user’s movements from a distance without the need for hand-held controls. The exercises used in the games were constructed from the evidence-based exercise programs Otago and FaME, designed to reduce falls among older people [11-13]. A short description of some of the exercises and games used in this study can be found in Multimedia Appendix 1. User performance was tracked by the MIRA system [9] and Microsoft Kinect.

The MIRA system [9] measures parameters such as (1) the number of exergames played including the frequency and duration, (2) progress statistics including game scores, distance, speed, and acceleration, and (3) overall movement activity during the games. This information is recorded by the MIRA system [9] and can also be remotely tracked by the physiotherapist and physician to monitor user performance. The user’s therapy schedule may be adjusted as required throughout their program.

To improve their physical function and reduce falls risk, older people need to be motivated to adhere to a therapy tailored exercise program. The systematic review and meta-analysis of Sherrington et al [14] suggest that an exercise program should run for at least two hours a week, for at least six months, to obtain a minimal effective exercise dose.

Motivation can be divided into intrinsic and extrinsic motivation according to the Self Determination Theory (SDT) [15]. Extrinsic motivation can be defined as behaviour in which people engage due to an objective consequence or separable outcome of that behaviour and intrinsic motivation is behaviour which people experience as interesting, satisfying and enjoyable [15]. Sweetser and Wyeth [16] developed the game flow model of player enjoyment (Table 1) that describes the intrinsic motivators that enhance users’ interest in playing computer games. This is known as the “flow” in which the person becomes immersed in the activity, losing a sense of self-consciousness and time. Enjoyment is further described as the fulfillment of 8 elements that create game enjoyment [16]. An overview of these 8 elements is presented in Table 1.

Previous literature has shown that intrinsic motivation is more important compared with extrinsic motivation to adhere to exercise for a longer period of time [17]. Nevertheless, it is unknown if and why older people are motivated to use exergames. Our goal was to explore the factors that influence the intrinsic motivation of older people to use MIRA-exergames [9] to improve their physical function and reduce fall risk.

**Table 1:** Definitions of the 8 elements of the game flow model of player enjoyment of Sweetser and Weyth [16].

Element	Definition
Concentration	The game should require some concentration, which can be defined as attention and workload. The more concentration the games requires, the more absorbing the game will be.
Challenge	The game should be challenging in which it matches the player’s skill level, a changeable difficulty level, and an appropriate pace. If the challenge is too high in comparison with the skills of the player, this might result in anxiety. If the challenge is to low in comparison with the skills of the players, this might result in apathy. Therefore, the challenge of the game should be in comparison with the skills of the player to make the player to enjoy and finally endure the game.
Player skills	According to Sweetser and Wyeth [16], the game should support the development and mastery of the skills of the player, otherwise enjoyment is not possible. Therefore, the challenge of the game should be correlated with the skills of the player. During this element, a good explanation of the game and the associated skills is essential. The element “player skills” can be related to the need of competence.
Control	This element can be related to the concept “autonomy” of the CET^a^. Control refers to a certain sense of control during the actions of the game. During the game, the player should be able to control the actual behaviors of their character in an intricate, effective, and easy way to explore the environment and to manipulate objects in the environment. This is important to carry out the player’s goals.
Clear goals	The game should have a clear goal. The goal should be made clear at the beginning of the game, this way the player knows from the start what to do. This can be done with a brief introduction movie. Each level should also contain multiple goals. These goals can be explained with so-called “briefings.”
Feedback	Appropriate feedback at appropriate time is important because concentration is only possible with immediate feedback. The player should receive feedback about his or her progress, and if the player does something wrong, he or she should receive feedback about how to do it right. Scores provide the player with positive feedback and encourages the player to improve.
Immersion	Immersion refers to deep but effortless experiences of the player regarding the game. During immersion, the player forgets about his or her surroundings and everyday life. Sometimes games are seen as an escape from the real world and everyday worries.
Social interaction	According to Sweetser and Wyeth [16], games should provide and create opportunities for social interaction. It can create competition, cooperation, and connection between players. However, social interaction can also interfere with immersion because other real people can be a link to the real world. Nevertheless, social interaction is a strong element for enjoyment, if people game for social interaction. The element “social interaction” can be related to the need relatedness of the SDT^b^.

^a^CET: Cognitive Evaluation Theory.

^b^SDT: Self Determination Theory.

## Methods

### Design

This study is a substudy of a larger study in which mixed-methods were used: interviews, Technology Acceptance Model (TAM) questionnaires, and 81 h of observation of the participants using the exergames were conducted. Data collection was conducted from May 2015 to July 2015. The interviews were undertaken at communal rooms in the assisted living facilities. Assisted living facilities are accommodations specifically designed for older people that usually consist of self-contained flats or bungalows with communal facilities, with a manager who oversees the maintenance of the facility (also known as sheltered housing or supportive housing for older people). People living in these facilities are able to receive extra care if required such as home help to assist with bathing.

**Figure 1:**
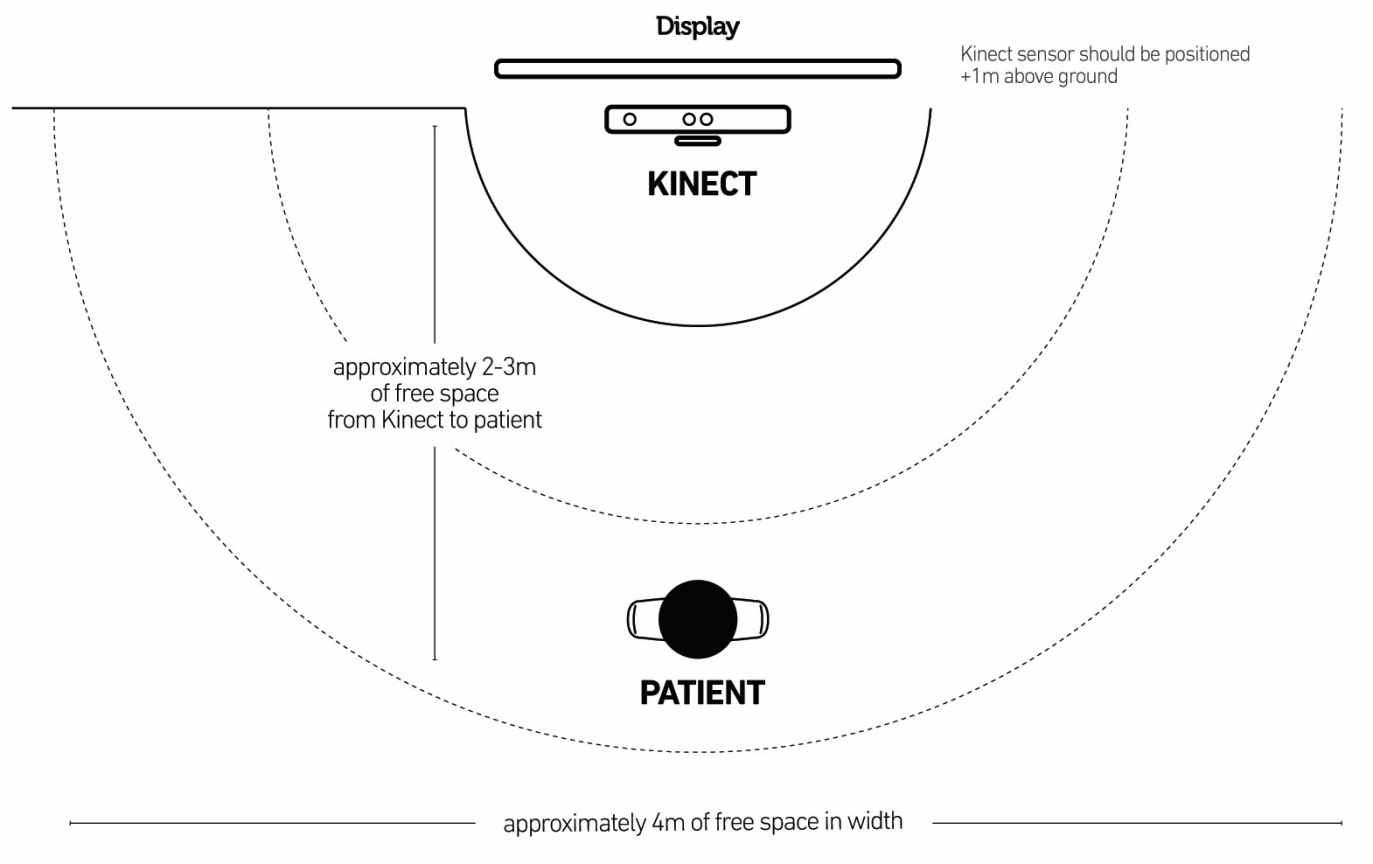
Mira-exergames system setup: The Microsoft Kinect camera is positioned directly in front of the screen. The user is positioned at 2 meters distance from the Kinect camera.

The exergame program was offered to the participants 3 times a week during a period of 6 weeks under the supervision of a physiotherapist. The participants were initially assessed by the physiotherapist who then tailored the exergames program according to the ability and needs of the participant. Each schedule began with calibration to ensure the user was positioned correctly for optimum performance and effective motion tracking and a tutorial that demonstrated how to perform the exercise that follows. One session took approximately 15 min on average for each participant, starting with 5 min and building up to 20-25 min. In one housing facility, the exergames were offered in the communal lounge area during a weekly coffee morning social event. In the second housing facility, the exergames were in a separate communal room where only the older participants met together in the room. The size of the groups varied from just 1 participant to 7 participants. The exergames are played individually. Participants mostly preferred to stay and watch other participants play the exergames, although some attended for only their session. The rooms had at least 2 m space to exercise safely, access to a large television screen with a laptop connection, and chairs for support during the exercises and for rest (see Figure 1). A transportable laptop with MIRA Rehab software [9] installed and the transportable Microsoft Kinect were connected by the physiotherapist to the television screen.

### Participants

In total, 12 older people in the age range of 59 to 91 years from 2 assisted living facilities in the North West of the United Kingdom, 1 physiotherapist with 3 months experience of using MIRA-exergames [9], and 1 manager of one of the assisted living facilities participated in this study. Older people were included in the program if they met the following criteria: (1) aged 59 years and older, (2) had used MIRA-exergames [9] for at least three weeks, (3) were able to speak and understand English language, (4) had the mental capacity to give informed consent, and (5) lived in an assisted living facility. Older people were excluded if they suffered from severe cognitive impairment. In addition, a qualified physiotherapist assessed the participant’s medical history, safety risk, and ability to engage in the exergames (both physically and cognitively). Table 2 provides an overview of the included participants.

**Table 2:** Participant characteristics and number of exergame sessions attended during 6 weeks.

Participant^a^	Age	Sex	Current medical condition(s)	Marital status	#sessions/6 weeks used
Ms Smith	67	Female	Depression with anxiety and bipolar disorder	Engaged	15
Mr Darcy	89	Male		Single or never married	17
Ms Colins	66	Female	Depression^b^	Divorced	13
Mr Crawford	63	Male	Disability right hand	Single or never married	6
Ms Price	80	Female		Widowed	14
Mr Rushworth	66	Male		Single or never married	16
Ms Bertram	79	Female	Mild cognitive impairment	Widowed	8
Mr Wickham	60	Male	Depression with anxiety	Engaged	15
Mr Willoughby	76	Male		Single or never married	18
Ms Bennet	81	Female	Depression	Widowed	3
Ms Dashwood	91	Female		Widowed	12
Mr Ferrars	59	Male		Single or never married	2

^a^The names of the participants are changed for anonymity.

^b^According to the DSM-IV criteria for depression and herself, this person suffers from a depression. However, this is not reported in her medical history.

### Measures

The interview guide consisted of questions related to the 8 elements of game enjoyment defined by Sweetser and Wyeth [16].

The TAM questionnaire consists of 4 subscales of statements of which 2 subscales are presented in this article, which are regarding the perceived ease of using MIRA-exergames [9] and the attitude toward MIRA-exergames [18]. The statements were categorized with a 7-point Likert scale.

### Data Analysis

The data were analyzed using constant comparative analysis. The interviews were transcribed, and open coding and axial coding was conducted by both researchers separately. Both researchers discussed their codes, and a coding scheme was developed from the different themes that emerge [19]. The interviews were selectively coded by both researchers separately and the coding was compared. After consensus was reached, the coding was analyzed using Atlas.ti coding program [20]. The questionnaire data were analyzed in SPSS Statistics 22. During 81 h of observation, notes were made of physical and verbal expressions until data saturation. Data saturation was reached after no new data and no new themes were observed [21].

### Ethical Approval

This study is conducted in full conformance with the principles of the Declaration of Helsinki, Good Clinical Practice, and within the laws and regulations of the United Kingdom and the European Union. Before commencing participant recruitment, approvals have been obtained from the University of Manchester and NHS Research Ethics Committees.

## Results

### Demographical Information

Interviews and TAM questionnaires were completed by 12 of the 19 participants approached. Of the 12 older participants, 5 suffered from mental or physical impairments, which may have influenced their motivation to participate in the exergames. Three participants, a 66-year-old woman, an 81-year-old woman, and a 60-year-old man suffered from depression. A 67-year-old woman suffered from both depression and bipolar disorder, and a 79-year-old woman suffered from mild cognitive impairment, but both were able to participate in the exergames. There were no adverse events reported during the study.

### Attitude Toward Exergames

In general, the majority (8 out of 12) of the older people had a positive attitude toward MIRA-exergames [9]. This was reported during the interviews but also confirmed by the TAM questionnaire. The third subscale of the questionnaire consisted of questions regarding the participant’s attitude toward the exergames. The mean score for attitude was 6.19 (SD=1.21; min: 2, max: 7; reliability alpha=.86) on the 7-point Likert scale, which indicated that the residents had a positive attitude toward the exergames.

### Enjoyment

In this study, 8 people said they enjoyed playing the exergames. A deep sense of enjoyment is described as a combination of 8 elements, see Table 1 [16]. Two older people did not enjoy playing the exergames. One of them felt that the exergames (and all games in general) were patronizing, and the other person had depression, which may have detracted from the enjoyment of the exergames. The remaining two of the 12 older people felt more neutral about the exergames or slightly enjoyed playing them. The results of the eight elements that define enjoyment in this study are described in detail below.

The first element, concentration, was required by all the participants to be able to focus on the screen and to do the associated movements. It was observed that the performance decreased when the participants were simultaneously having a conversation (known as dual tasking).

The second element, challenge, was experienced by 10 participants because they experienced the exergames as something new, which they have never done before. The older participants experienced the exergames as mentally challenging because they had to remember the games and what they had to do during the games. For some, it was physically challenging to be able to undertake the movements. The participants appeared to challenge themselves to improve on their performance each session. To start undertaking the exergames was also experienced as a challenge that resulted in the participant feeling proud and a sense of achievement. Finally, it appeared that some exergames were experienced as more challenging compared with others.

The third element was a clear goal of the game. According to the older people, the general goal of using the exergames was to win as many points as possible and to do more physical exercise. The goal of the games itself were not clear for 5 of the 12 older people even when they played these games often.

The fourth element, feedback, was provided through the provision of scores and instructions given by the physiotherapist. The exergames also provide instant feedback. This included success or failure sounds, written text for encouragement and to indicate correct position for the sensor, green color code to indicate correct movement, and red for incorrect movements. At the end of each session the motion tracking analysis give feedback about points won, duration of session, range of movement, number of repetitions, average speed and acceleration that can be viewed by the participant and the physiotherapist. According to the participants, additional feedback about their progress from the physiotherapist was important. Six older participants were also doubtful if they would be able to play the exergames on their own without the support of the physiotherapist. Five residents believed they would be able to use the exergames by themselves after doing it together with the physiotherapist.

The fifth element, immersion, was experienced by 9 residents. Immersion appeared to create a positive experience among some of the residents because the exergames were found to provide an escape from reality.

The sixth element, control of the character, was experienced by all the residents. For example, when they moved their arm up, the character in the game went up.

It was observed that all residents had the skills to play the exergames, which refers to the seventh element. The residents reported that the difficulty-level of the exergames set by the physiotherapist was correct for them. The TAM questionnaire also confirmed the perceived ease of use as easy or just right. The first subscale of the TAM questionnaire consists of statements regarding perceived ease of use, which scored on average 5.60 (SD=1.36; min: 2, max: 7; reliability alpha=.84) on the 7-point Likert scale, which indicated that the exergames were found to be easy to use with the current set-up.

The final element, social interaction, appeared also to be one of the main findings in this study. During the observations it was noticed that the games themselves do not provide social interaction because the user has to do the exercise alone. However, the exergames were set up in communal rooms at the support housing facilities that made social interaction with other people possible before and after doing the exergames. The group size varied from 1 participant to up to 7 participants taking part in an exergame session. The participants played the exergames individually but were observed by the other attending participants. During the interviews and observations, it was clear that the social interaction encouraged the older participants to undertake the exergames, for example, it created competition between people that resulted in further motivation to improve. Another participant mentioned that it created opportunities to learn from and interact with each other. Seven older people were doubtful if they would do the exergames alone at their apartment, since they felt motivated by the group to do the exergames because others were doing them as well. Nevertheless, the findings also indicate that the social interaction might decrease someone’s confidence because, for example, others might have higher scores. One participant with lower scores mentioned that others might start thinking something is wrong with her because she has lower scores. Furthermore, 2 participants mentioned they felt watched or judged by other people who were also in the room, which resulted in an uncomfortable feeling. These feelings might decrease the motivation to do the exergames. However, the older participants who mentioned these negative influences of the social interaction preferred in the end the set-up with social interaction. It appeared that the participants were not aware of their surroundings after undertaking a few exergame sessions.

During the observations, it appeared that not only was social interaction with other older people important, but also social interaction with the instructors of the exergames. The conversations with the instructors seemed to have a positive influence on the older participants because during informal conversations the older participants regularly thanked the instructors for being present and said they enjoyed their company. This observation was also confirmed by one of the participants during the interview.

### Confidence

During the interviews and observations it appeared that besides enjoyment, confidence was also an important factor that influenced the intrinsic motivation of older people to use the exergames. This confidence was related to physical strength, mental capacity, and the social setting. The confidence in having physical strength appeared to be related to fear of falling. Older people reported that they became more confident in their physical function after doing the exergames, which might have also reduced their fear of falling. Older people could also be assumed to have gained confidence in relation to their cognition because they were able to do something new and develop new skills. There was also a confidence noted in the social interaction. Additionally, 2 older people did not feel comfortable doing the exergames in front of others; however, this changed after undertaking a few sessions in the social setting. This indicates that confidence related to social interaction may increase by undertaking the exergames in a social setting. The physiotherapist and the warden also confirmed that they saw an increase in confidence in relation to the social setting.

The instructors also appeared to have a large influence on the confidence of their clients. It was observed that the instructors made a lot of positive comments toward the older people. The instructors said things like “you can do it,” “well done,” “good job,” “you are really improving,” and “I am impressed.” These positive comments may have boosted the confidence of residents. Nevertheless, it should be considered that a coach is an extrinsic motivator who strengthens the intrinsic motivation. Coaching is mentioned as an intrinsic motivator in this study because it has a strong influence on the intrinsic motivation of the resident and does not influence the desired outcome of the participants for doing the exergames.

## Discussion

### Principal Findings

This study investigated the factors that may influence the intrinsic motivation of older people to use MIRA -exergames [9] to improve their physical function and reduce fall risk. The findings of this study show that most of the older participants enjoyed playing the exergames. Previous qualitative studies on exergames confirm that exergames in general are enjoyable [22-25]. These previous studies and this study demonstrate that exergames may be promising tools to enable physical exercises to be more enjoyable. However, it should be taken into account that not everyone may be interested or willing to use exergames to exercise and improve their physical function.

In general, all 8 elements, which define enjoyment according to the game flow model of Sweetser and Wyeth [16], were fulfilled for the older participants who enjoyed playing the exergames in this study. The systematic review by Boyle et al [26] regarding engagement in computer games describes elements for game enjoyment: “Flow, the best known term for describing the enjoyable subjective experiences of playing games, has a strong focus on cognitive features relating to the task such as challenge, concentration, goals and feedback.” The study of Boyle et al [26] confirms the importance of concentration, challenge, clear goals, and feedback in games to make enjoyment possible, which are also the first 4 elements discussed above of the game flow model of Sweetser and Wyeth [16]. Furthermore, the older people in this study experienced the exergames as challenging. The importance of challenge in games is also acknowledged by Jackson and Csikszentmihalyi [27], who described game flow as a balance between the skills of the player and challenges in the game. Jackson and Csikszentmihalyi [27] define this balance as “the golden rule of flow” in games.

During the exergame program, feedback was provided by scores and during or post game screen feedback such as “congratulations” and “well done.” Additional feedback was provided by the instructors of the exergames. The findings indicate that an individual approach is required during which the coaching of the instructors is an important factor to increase intrinsic motivation. Positive comments of the exergame instructors toward the older users appear to improve the intrinsic motivation. Furthermore, tailored coaching that corresponds with the individual needs and wishes of the older person is essential. The importance of coaching regarding motivation was also acknowledged by Pelletier et al [28], who stated “those providing feedback of competence and a clear structure or rationale for doing an activity, foster self-determined forms of motivation *.”*

Immersion was experienced by most of the older people because playing the exergames was experienced as an escape from reality and/or problems. These experiences are also acknowledged by Jennett et al [29], who describe that games provide the possibility to “lose” one self in the world of the game. Jennett et al [29] state that *“* Immersion is often viewed as critical to game enjoyment, immersion being the outcome of a good gaming experience.”

The importance of social interaction is one of the main findings in this study. Brox et al [30] also investigated the importance of social interaction in exergames. They acknowledged that older people tend to be more homebound, and many of them suffer from loneliness and a lack of physical exercise. Therefore, Brox et al [30] recommend that persuasive Web-based social-exergames should be investigated further to increase the social interaction and exercise among older people. This study also acknowledges the importance of social interaction during the exergames. Therefore, this study also recommends a set-up of the exergames which makes social interaction with other people and instructors possible.

Besides enjoyment, another important intrinsic motivator raised during the interviews was the confidence of the participants. It appeared that the confidence of the participants increased after undertaking the exergames. The confidence of the residents who played the exergames was related to their perceptions of improved physical strength and mental capacity, and social interaction. Schutzer and Graves [31] investigated the barriers and motivations of older people to undertake exercise. They mention “self-efficacy is consistently identified as an important determinant of exercise behavior in various populations and in many types of behavioral learning throughout the scientific literature.” The concept “confidence” in this study can be correlated to self-efficacy. Schutzer and Graves [31] state that “The stronger one’ self-efficacy expectations and outcomes, the more likely the individual will initiate and persist with a specific behavior. Barring health factors, self-efficacy exerts a consistently powerful influence on the exercise behavior of older adults *.* ” The findings of Schutzer and Graves [16] confirm the influence and importance of confidence in the motivation of older people to use MIRA-exergames [9].

### Strengths and Limitations

The use of mixed methods increases the validity of the findings in this study. The interviews, questionnaires, and 81 h of observations in general were in agreement and confirmed the findings. Nevertheless, a limitation of this study is that not all the 19 older people who joined the exergames program participated in this study. Of these, 7 older people declined to participate in this study because of various reasons (too onerous, too busy, or unwell). This might indicate some selection bias in this study. This selection bias might indicate overestimation of the motivation of older people toward exergames because most of the participants in this study adhered to the exergame program for a longer period of time.

### Further Research

Several older people believed the MIRA-exergames [9] helped them to stay mentally active. However, further research is needed to investigate whether the exergames also stimulate cognitive function, and if so, whether exergames can reduce the risk of cognitive decline.

The findings of this study showed that social interaction appeared to be an important factor for the motivation of older people to do the exergames, which was also confirmed by the study of Brox et al [30]. Further research is recommended that investigates the effects of a social set-up of MIRA-exergames [9] on the mental and physical well-being and loneliness of older people. This way, the exergames could have different objectives to reach besides fall prevention.

### Conclusions

In conclusion, MIRA-exergames [9] appear to be a promising tool to improve the physical functions of older people and reduce their fall risk because older people may be intrinsically motivated to use them for a longer period of time. The implementation of the falls prevention exergames in the daily lives of older people may reduce health care costs related to falls among older people. Furthermore, wider uptake and increased adherence to exergames, and thus, increased physical activity might also reduce the risk of other diseases such as vascular disease and osteoporosis, which could lead to further reduced health care costs and improve the well-being and quality of life among older people. Larger studies are required to investigate these hypotheses.

## Appendix

Multimedia Appendix 1 Example of exergames for a falls prevention rehabilitation schedule (supervising physiotherapist prescribes easy, moderate, or hard levels; duration; and rest periods according to assessed level of ability).

## References

[1] United Nations, Department of Economic and Social Affairs, Population Division (2013). UN.

[2] Kinsella K, He W (2009). Census.

[3] World Health Organisation (2015). WHO.

[4] Rubenstein LZ (2006). Falls in older people: epidemiology, risk factors and strategies for prevention. Age and ageing.

[5] Deandrea S, Lucenteforte E, Bravi F, Foschi R, La Vecchia C, Negri E (2010). Risk factors for falls in community-dwelling older people: a systematic review and meta-analysis. Epidemiology.

[6] World Health Organization (2008). WHO.

[7] Den Hertog P, Stam C, Valkenberg H, Bloemhoff A, Panneman M, Klein Wolt K (2013). Veiligheid.

[8] Gillespie LD, Robertson MC, Gillespie WJ, Sherrington C, Gates S, Clemson LM, Lamb SE (2012). Interventions for preventing falls in older people living in the community. Cochrane Database Syst Rev.

[9] MIRA Rehab Limited MIRA Rehab.

[10] Calin A, Stanmore E (2015). Software.

[11] Campbell AJ, Robertson MC, Gardner MM, Norton RN, Tilyard MW, Buchner DM (1997). Randomised controlled trial of a general practice programme of home based exercise to prevent falls in elderly women. BMJ.

[12] Thomas S, Mackintosh S, Halbert J (2010). Does the 'Otago exercise programme' reduce mortality and falls in older adults?: a systematic review and meta-analysis. Age Ageing.

[13] Skelton D, Dinan S, Campbell M, Rutherford O (2005). Tailored group exercise (Falls Management Exercise -- FaME) reduces falls in community-dwelling older frequent fallers (an RCT). Age Ageing.

[14] Sherrington C, Whitney JC, Lord SR, Herbert RD, Cumming RG, Close JC (2008). Effective exercise for the prevention of falls: a systematic review and meta-analysis. J Am Geriatr Soc.

[15] Deci EL, Ryan RM (1985). Intrinsic motivation and self-determination in human behavior. Springer Science & Business Media.

[16] Sweetser P, Wyeth P (2005). GameFlow: a model for evaluating player enjoyment in games. ACM CIE.

[17] Richard M, Christina MF, Deborah LS, Rubio N, Kennon MS (1997). Intrinsic motivation and exercise adherence. Int J Sport Psychol.

[18] Chuttur MY (2009). Overview of the technology acceptance model: origins, developments and future directions. Am J Syst Software.

[19] Boeije H (2002). A purposeful approach to the constant comparative method in the analysis of qualitative interviews. Qual Quant.

[20] Scientific Software Development GmbH ATLAS.ti.

[21] Fusch PI, Ness LR (2015). Are we there yet? Data saturation in qualitative research. Qual Rep.

[22] Fitzgerald D, Trakarnratanakul N, Smyth B, Caulfield B (2010). Effects of a wobble board-based therapeutic exergaming system for balance training on dynamic postural stability and intrinsic motivation levels. J Orthop Sports Phys Ther.

[23] Osorio G, Moffat DC, Sykes J (2012). Exergaming, exercise, and gaming: sharing motivations. Games Health J.

[24] Rosenberg D, Depp CA, Vahia IV, Reichstadt J, Palmer BW, Kerr J, Norman G, Jeste DV (2010). Exergames for subsyndromal depression in older adults: a pilot study of a novel intervention. Am J Geriatr Psychiatry.

[25] van Diest M, Lamoth CJ, Stegenga J, Verkerke GJ, Postema K (2013). Exergaming for balance training of elderly: state of the art and future developments. J Neuroeng Rehabil.

[26] Prey JE, Woollen J, Wilcox L, Sackeim AD, Hripcsak G, Bakken S, Restaino S, Feiner S, Vawdrey DK (2014). Patient engagement in the inpatient setting: a systematic review. J Am Med Inform Assoc.

[27] Jackson SA, Csikszentmihalyi M (1999). Flow in sports.

[28] Pelletier LG, Fortier MS, Vallerand RJ, Tuson KM, Briere NM, Blais MR (1995). Toward a new measure of intrinsic motivation, extrinsic motivation, and amotivation in sports: The Sport Motivation Scale (SMS). J Sport Exerc Psychol.

[29] Jennett C, Cox AL, Cairns P, Dhoparee S, Epps A, Tijs T, Walton A (2008). Measuring and defining the experience of immersion in games. Int J Hum Comput Stud.

[30] Brox E, Luque LF, Evertsen GJ, Hernández JEG (2011). Exergames for elderly: Social exergames to persuade seniors to increase physical activity.

[31] Schutzer KA, Graves BS (2004). Barriers and motivations to exercise in older adults. Prev Med.

