# Supplementary material for: Motivational Determinants of Exergame Participation for Older People in Assisted Living Facilities: Mixed-Methods Study
Source: J Med Internet Res. 2017 Jul 6;19(7):e238. doi: 10.2196/jmir.6841 (PMC5519828; doi:10.2196/jmir.6841)
Supplement: Multimedia Appendix 1 [file jmir_v19i7e238_app1.pdf]

Name of exercise	Type of exercise	Compatible game for the specific exercise	Game instruction
Functional reach	Upper and lower limb coordination and flexibility: The person is instructed to use their designated arm to reach to pick up and move the object.	Grab 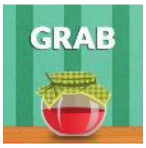	The player should pick up the objects and place them carefully on the shelves without dropping them.
Squats	Lower limb strength: The person is instructed to bend their knees, lower the torso, and then return to the upright position, keeping their trunk upright. The heels must not be lifted during the movement.	Atlantis 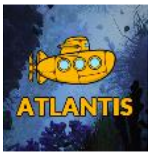	The player has to drive the submarine and discover the aquatic treasures while avoiding threats.
Knee flexion and extension	Lower limb balance: The person is instructed to stand up tall, bend the knee by bringing the foot toward the bottom, keeping the knee in line with the hip, and then return to starting point.	Izzy the bee 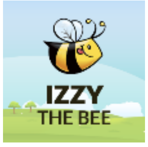	The player has to move the bee to gather nectar and bring it safely to the beehives, while avoiding the other bugs.
Sit to stand	Lower limb strength and balance: The person should sit tall near the front of a straight-backed firm chair with no armrests and be instructed to stand up and sit down with the arms folded across the chest.	Powerhouse bid 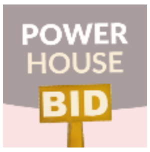	The player takes part in an auction and has to stand and sit at required times to bid for the auctioned items.

Hip abduction	Balance and lower limb strength: The person is instructed to stand up straight with legs together and knees slightly bent. Move designated leg out and away from your body with control before returning to original position lift. The leg should be straight and the lifting should be done to the recommended range of motion.	Catch<br>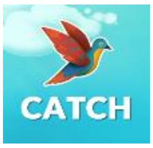	The player will have to catch all items before they disappear.
